# Improvement of Chronic Lateral Knee Pain Through Osteopathic Manipulative Treatment Targeting Anterior Fibular Head Somatic Dysfunction: A Case Report

**DOI:** 10.7759/cureus.105266

**Published:** 2026-03-15

**Authors:** Abraham E Libman, Mikhail Volokitin, Julia Speiser, Lianna Rocchio, Tzipora Benyaminov, Nagma Tai, Shazib Naseer

**Affiliations:** 1 Osteopathic Manipulative Medicine, Touro College of Osteopathic Medicine, New York, USA; 2 Internal Medicine, Touro College of Osteopathic Medicine, New York, USA

**Keywords:** anterior fibular head, chronic knee pain, fibular head dysfunction, high velocity low amplitude, knee biomechanics, musculoskeletal pain, nonoperative management, osteopathic manipulative treatment, proximal tibiofibular joint, somatic dysfunction

## Abstract

Chronic knee pain in young adults is frequently managed through conservative modalities, yet persistent dysfunction often leads to early orthopedic referral and eventual surgical intervention. This case describes a 26-year-old female medical student with nearly a decade of debilitating right lateral knee pain that persisted despite bracing, physical therapy, and corticosteroid injections.

In the setting of valgus alignment and chronic symptoms, surgical options were discussed during orthopedic evaluation, but the patient pursued osteopathic manipulative treatment prior to reconsidering operative management. This patient also presented with multiple chronic comorbidities, including Hashimoto's thyroiditis, type 2 diabetes mellitus, and a history of obesity, all of which are known to increase mechanical stress and systemic inflammation contributing to musculoskeletal pain. Despite medical management for these metabolic and autoimmune disorders, her knee symptoms persisted and progressively worsened over several years.

Osteopathic structural examination identified a right anterior fibular head somatic dysfunction. High-velocity, low-amplitude manipulation was performed, resulting in immediate and substantial pain reduction from 9/10 to 4/10, with near-complete relief over the following 24 hours. Continued myofascial and muscle-energy treatments were administered to maintain pain reduction and function for weeks thereafter. This case highlights the potential of targeted osteopathic manipulative treatment as a non-surgical, low-risk alternative for chronic knee pain in young adults and supports broader consideration of fibular head biomechanics in musculoskeletal diagnosis and management.

## Introduction

Chronic knee pain represents a major cause of disability in young adults, with increasing prevalence due to obesity, sports injury, and metabolic disease [1]. Traditional management with nonsteroidal anti-inflammatory drugs (NSAIDs), corticosteroid injections, and physical therapy often yields incomplete or transient relief, and many patients referred for orthopedic evaluation ultimately do not proceed to surgery [2,3]. Obesity and metabolic disorders contribute to increased mechanical load and systemic inflammation, accelerating degenerative changes and complicating the nonoperative management of knee pain [4]. In such patients, osteopathic principles emphasize the comprehensive evaluation of biomechanical contributors to pain, including alignment and somatic dysfunction, as part of a noninvasive treatment strategy [5]. When structural imaging reveals degenerative changes, orthopedic surgeons may recommend corrective osteotomy or total knee arthroplasty; however, joint replacement longevity and revision surgery risks often render such procedures undesirable in younger patients [6]. In patients with chronic knee pain, evaluation of global lower-extremity alignment is therefore critical for selecting the optimal form of management.

Obesity and metabolic disorders significantly increase the mechanical load on lower-extremity joints and can accelerate the development of degenerative knee changes [7]. In addition, endocrine and autoimmune conditions such as hypothyroidism and diabetes mellitus can impair tissue repair, alter connective tissue elasticity, and contribute to chronic inflammation [8]. These systemic factors not only predispose patients to earlier onset of osteoarthritis but can also reduce responsiveness to conventional treatments such as corticosteroid injections and physical therapy. Consequently, patients with complex metabolic backgrounds may benefit from a more integrative approach, including osteopathic manipulative treatment (OMT), which targets both the biomechanical and physiologic contributors to chronic pain [9].

Osteopathic physicians evaluate musculoskeletal complaints through the assessment of somatic dysfunction, including altered function of related skeletal, arthrodial, and myofascial structures. The proximal tibiofibular joint and fibular head are key contributors to knee stability and ankle-knee biomechanics. Restriction of fibular head anterior-posterior glide may increase lateral knee tension and generate pain patterns mimicking meniscal or ligamentous pathology [10]. Restoration of physiologic motion through OMT may alleviate pain and restore function without invasive intervention.

## Case presentation

A 26-year-old female medical student with a history of Hashimoto's thyroiditis, type 2 diabetes mellitus, and iron deficiency anemia presented with chronic right knee pain of nearly a decade's duration. The patient had a long-standing history of weight fluctuations, having reached approximately 300 pounds in high school before losing significant weight through lifestyle modifications, maintaining a weight of 165-180 pounds during her undergraduate years. She began semaglutide (Ozempic) two years prior for glycemic and metabolic control, achieving modest weight reduction. Despite these medical and lifestyle efforts, she continued to experience debilitating knee pain that limited her ability to exercise regularly.

The patient's symptoms began during adolescence following a snapping sensation and swelling during physical activity. The pain persisted through her undergraduate years despite conservative management and worsened over time, ultimately interfering with sleep and prolonged standing. Over the past two years, the patient reported progressively increasing pain with associated difficulty ambulating, limiting prolonged walking and standing. She later pursued osteopathic evaluation during her medical training after continued symptoms and out of concern about the potential need for future surgical intervention. Overall, the patient experienced chronic symptoms for approximately 10 years, underwent orthopedic imaging and consultation during her undergraduate years, and later received osteopathic evaluation and treatment during medical school.

**Figure 1:**
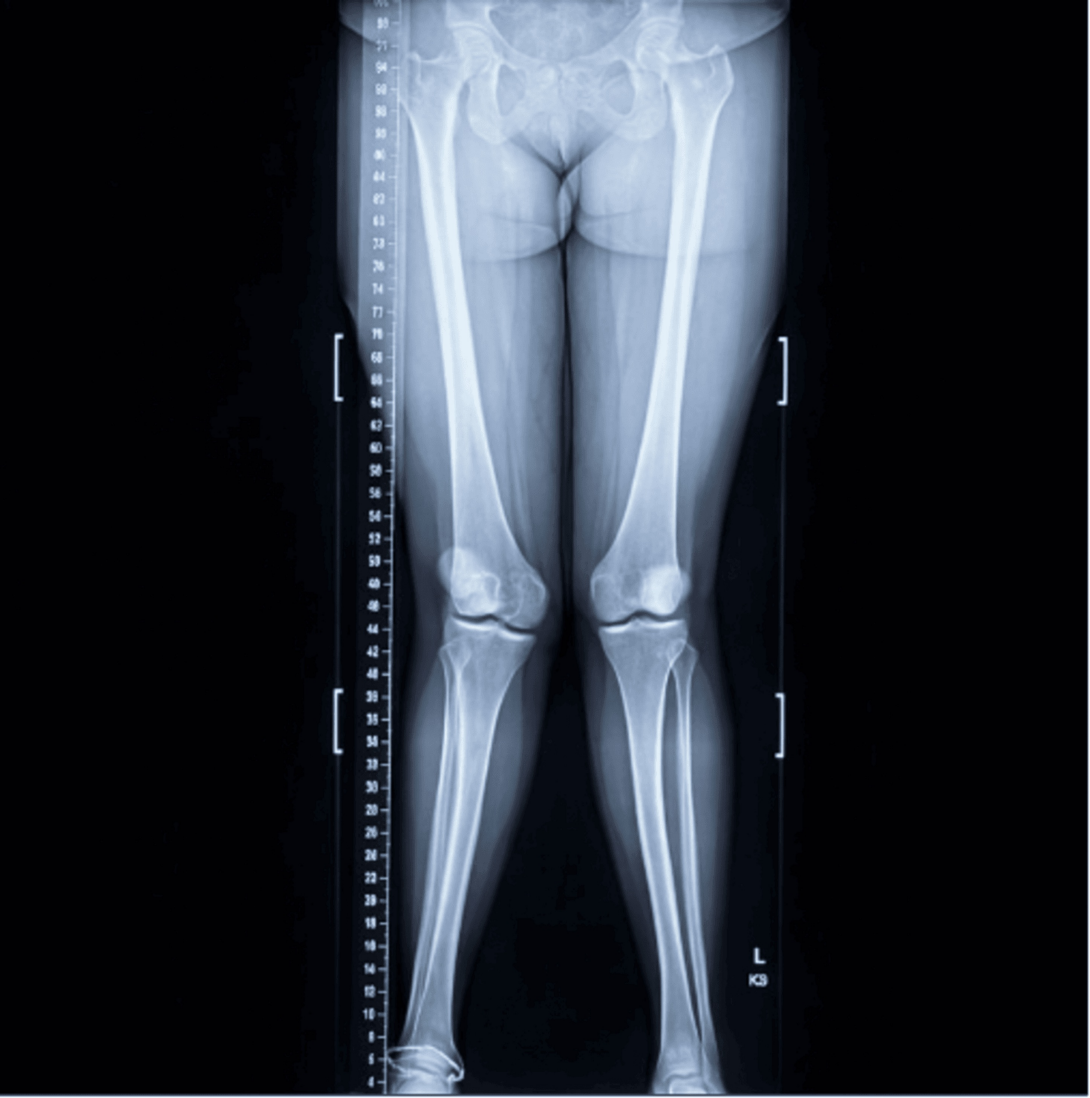
Full-length standing anteroposterior lower-extremity radiograph demonstrating the patient's genu valgum (knock-knee) deformity

Weight-bearing full-length radiographs obtained during the patient's prior orthopedic evaluation were used to assess mechanical axis deviation and tibiofemoral alignment. The patient's full-length standing anteroposterior radiograph demonstrating valgus knee alignment consistent with genu valgum is shown in Figure 1. All radiograph and magnetic resonance imaging (MRI) reports and interpretations referenced below were provided by a board-certified radiologist.

The patient's valgus knee alignment, which is consistent with genu valgum (knock-knee deformity), entails the mechanical axis deviating laterally at the level of the knee bilaterally. Mild asymmetry in the tibiofemoral joint space is noted, with relatively decreased compartment spacing on the right compared with the contralateral side. A calibrated ruler is present in Figure 1 to facilitate the assessment of coronal plane alignment and limb length.

**Figure 2:**
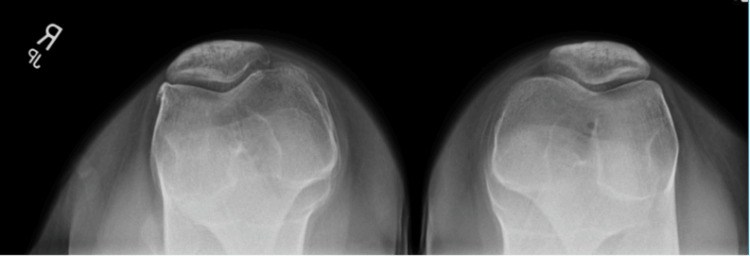
Bilateral axial (sunrise) radiographs of the patellofemoral joints showing no acute fractures

The patient's pain began in high school after a "snapping" sensation occurred during an exercise in her physical education class, followed by swelling and persistent discomfort. She denied any specific acute injury beyond the initial incident, but noted persistent lateral knee tenderness and difficulty standing for long periods. Initial imaging revealed no acute fracture, as shown in Figure 2. Conservative management with bracing and rest provided transient relief.

Figure 2 shows a bilateral axial (sunrise) radiograph of the patient's knees, demonstrating preserved patellofemoral alignment without evidence of acute fracture, dislocation, or significant patellar maltracking. Joint spaces appear symmetric, with no focal osteochondral defect or advanced degenerative change identified. These findings further support the absence of primary patellofemoral pathology contributing to the patient's lateral knee pain.

Pain severity was assessed using a Numeric Rating Scale (NRS) ranging from 0 (no pain) to 10 (worst possible pain). During her undergraduate years, the patient's pain worsened as she developed progressive, throbbing lateral knee pain (rated 9-10/10). It was exacerbated by standing or walking and interfered with sleep. Orthopedic evaluation revealed degenerative changes attributed to mechanical strain and weight history [4,7]. Corticosteroid injections and physical therapy temporarily reduced the pain to 6-7/10. Repeated intra-articular corticosteroid injections provided short-term symptomatic relief, but benefits were transient and diminished in duration with subsequent injections, limiting their utility as a long-term strategy for chronic knee pain. Subsequent imaging and orthopedic evaluation led to the discussion of possible future surgical options, including corrective osteotomy and earlier-than-typical arthroplasty if symptoms progressed; however, the patient preferred to pursue nonoperative options [6].

**Figure 3:**
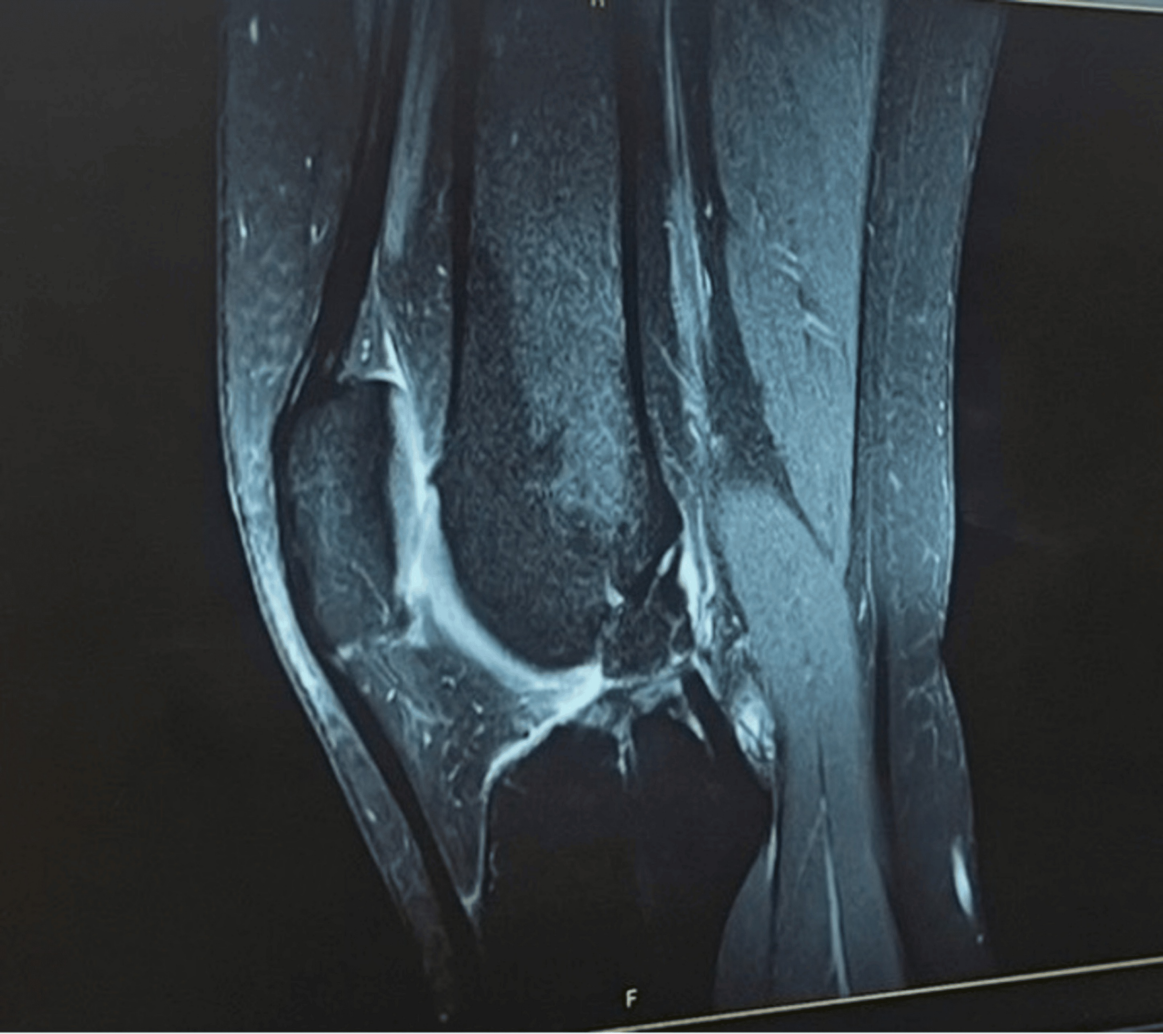
Coronal MRI of the right knee demonstrating lateral compartment findings MRI: magnetic resonance imaging

Figure 3 shows a coronal MRI of the right knee, demonstrating preserved osseous alignment without evidence of acute fracture, ligamentous rupture, or meniscal tear. Mild signal variation is observed along the lateral compartment near the proximal tibiofibular articulation, without a discrete intra-articular lesion. In the setting of persistent lateral knee pain and otherwise unremarkable imaging, these findings supported the consideration of a biomechanical rather than primary structural etiology.

**Figure 4:**
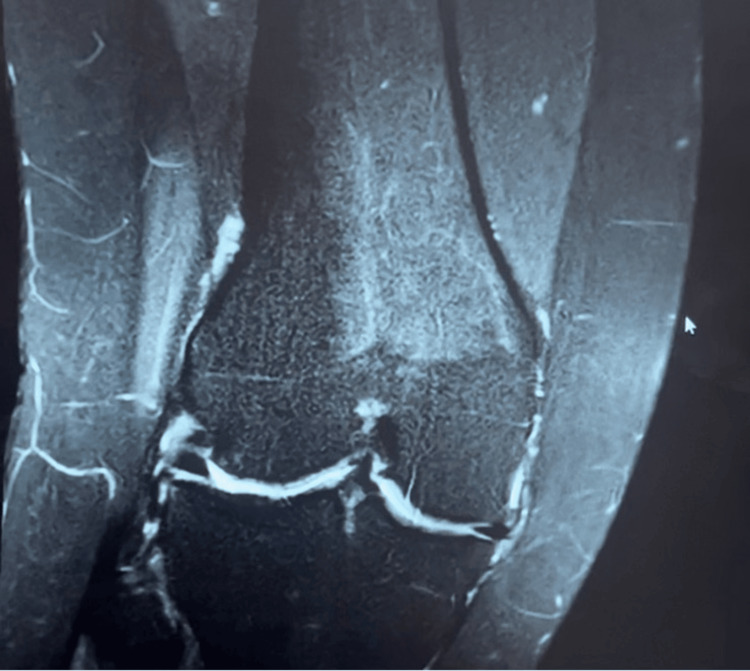
Coronal MRI of the right knee demonstrating lateral compartment joint space narrowing MRI: magnetic resonance imaging

Figure 4 shows a coronal MRI of the right knee obtained during the patient's orthopedic evaluation for persistent symptoms prior to osteopathic treatment. The image demonstrates the lateral compartment and proximal tibiofibular region with relative narrowing of the lateral joint space compared to adjacent compartments. No acute fracture, ligamentous disruption, or full-thickness meniscal tear is identified. The observed reduction in joint space, in the absence of discrete intra-articular pathology, was interpreted in the clinical context as potentially reflective of altered biomechanics, rather than a primary structural lesion.

**Figure 5:**
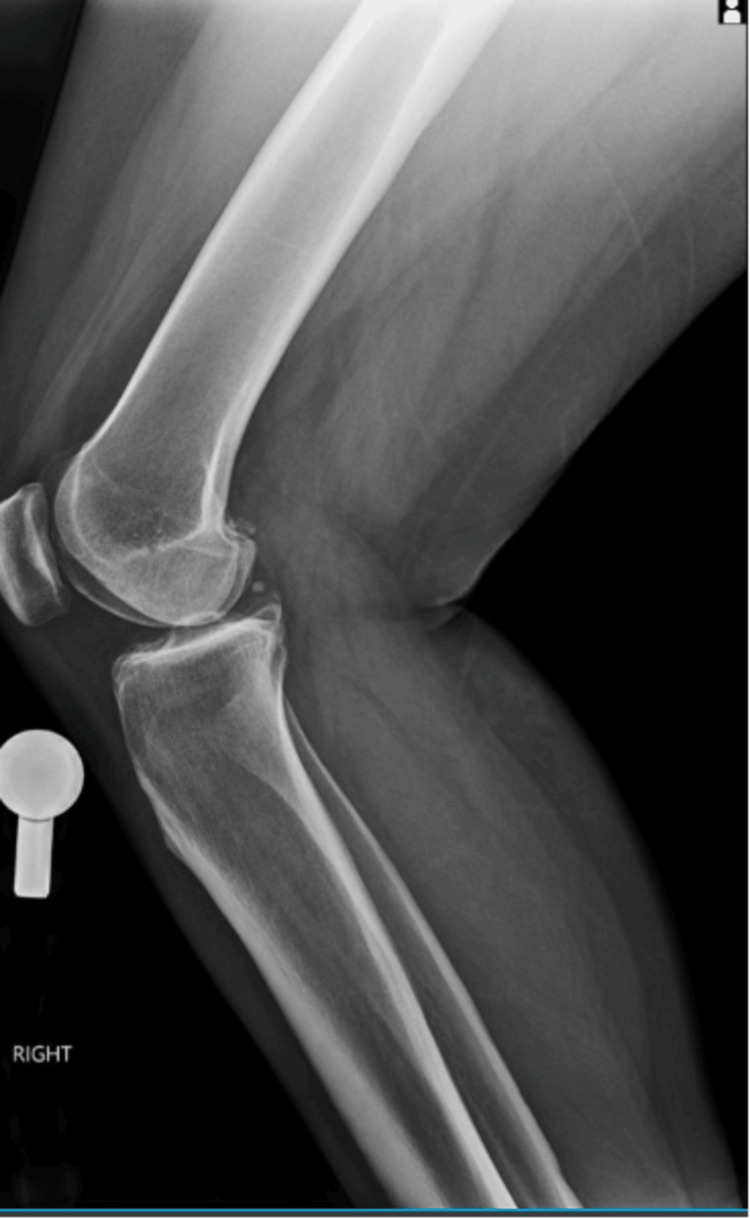
Lateral radiograph of the right knee demonstrating preserved sagittal alignment

Figure 5 shows a lateral radiograph of the right knee demonstrating preserved sagittal alignment of the femorotibial and patellofemoral articulations. No acute fracture, dislocation, or large joint effusion is identified. Joint spaces appear maintained on the lateral view, with no advanced degenerative changes noted. These findings further support the absence of gross structural pathology contributing to the patient's lateral knee pain.

When the patient began medical school, her increasing understanding of musculoskeletal anatomy deepened her curiosity about the structural contributors to her chronic pain. As a student of osteopathic medicine, she expressed interest in exploring OMT as a last resort before reconsidering the surgical options that had previously been advised by orthopedic specialists.

She later presented for osteopathic evaluation during her medical training. Osteopathic structural examination revealed restricted posterior glide and free anterior motion of the right fibular head, consistent with anterior fibular head somatic dysfunction [10]. Attempted glide of the fibular head reproduced the patient's focal lateral knee tenderness. Standing anteroposterior and weight-bearing posteroanterior knee radiographs demonstrated asymmetric lateral compartment narrowing on the right, supporting a chronic mechanical contribution and consistent with focal proximal tibiofibular dysfunction on examination.

**Figure 6:**
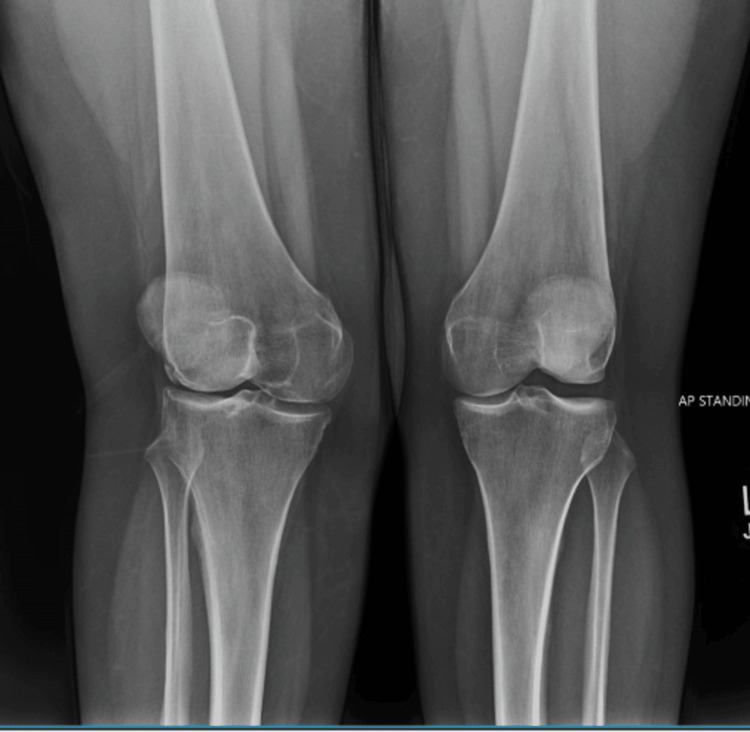
Standing anteroposterior radiograph of both knees demonstrating asymmetric joint space narrowing

Figure 6 shows a standing anteroposterior radiograph of both knees demonstrating relative narrowing of the lateral joint space in the right knee compared with the contralateral side. Overall osseous alignment is preserved, with no acute fracture or dislocation identified. The asymmetric joint space narrowing, in the absence of advanced degenerative changes, supports a biomechanical contribution to the patient's lateral knee symptoms and correlates with osteopathic structural findings involving the proximal tibiofibular region.

**Figure 7:**
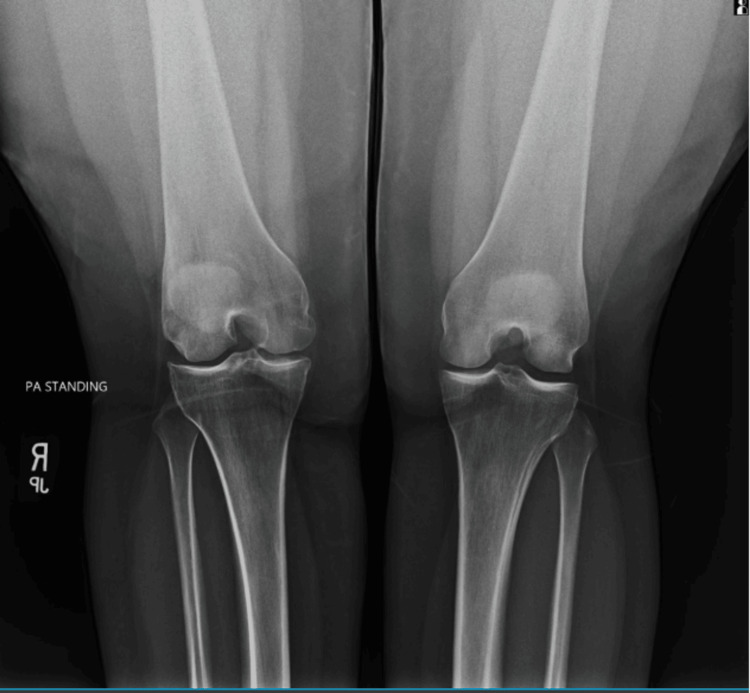
Weight-bearing posteroanterior radiograph of both knees demonstrating asymmetric lateral compartment joint space narrowing

Figure 7 shows a weight-bearing posteroanterior radiograph of both knees, demonstrating relative narrowing of the lateral compartment joint space of the right knee compared with the contralateral side. Overall bony alignment is preserved, with no evidence of acute fracture, dislocation, or advanced degenerative change. The asymmetric joint space narrowing observed under physiologic load further supports a functional and biomechanical contribution to the patient's lateral knee symptoms, correlating with osteopathic structural findings involving the proximal tibiofibular region. 

Written informed consent for the publication of this case report and associated imaging was obtained from the patient. This case report was prepared in accordance with the CARE (CAse REport) guidelines for transparency and completeness in clinical case reporting.

OMT intervention

A comprehensive osteopathic structural examination was conducted. Palpation revealed tenderness over the lateral aspect of the right knee. Fibular head motion was assessed using a proximal tibiofibular joint glide test in which the examiner stabilizes the tibia while applying anterior and posterior translational forces to the fibular head to evaluate joint mobility. Restricted posterior glide of the fibular head was noted along with mild hypertonicity of the peroneus longus and biceps femoris muscles. Tissue texture changes were noted over the lateral collateral ligament and proximal fibular head, with subtle edema suggesting chronic irritation. Based on these findings, a diagnosis of right anterior fibular head somatic dysfunction was made.

The risks, benefits, and alternatives of OMT, including high-velocity, low-amplitude (HVLA), were reviewed, and the patient provided verbal consent. No contraindications to HVLA (including suspected fracture, acute instability, or neurovascular compromise) were identified. The physician positioned the patient supine with her right knee flexed to approximately 90 degrees. The fibular head was engaged posteriorly with the operator's thenar eminence, while the ankle was plantarflexed and inverted to place tension through the fibular articulation. A HVLA thrust was delivered anterior-to-posterior to restore the posterior glide of the fibular head, producing an audible release in the form of a "pop" [10]. The patient reported immediate pain reduction and increased knee mobility, reflected clinically by improved tolerance for ambulation and prolonged standing without recurrence of the prior catching sensation, as well as reduced pain on fibular head spring testing. HVLA was selected due to a clear restrictive barrier at the proximal tibiofibular articulation with reproducible pain provocation on spring testing [10,11].

Following the initial treatment, the physician demonstrated to the patient and her partner basic myofascial and muscle energy techniques targeting the peroneal, gastrocnemius, and hamstring musculature to support ongoing maintenance [11,12]. Subsequent sessions incorporated myofascial release and muscle energy techniques directed at the surrounding peroneal and hamstring musculature.

Outcomes

Immediately following the HVLA treatment, the patient's pain decreased from 9/10 to 4/10, and she ambulated without the lateral tension or catching sensation that had been plaguing her consistently for several years. Post-treatment reassessment demonstrated decreased tenderness over the proximal fibular head and improved posterior glide on spring testing, with resolution of the previously reproducible focal lateral knee pain provocation. She reported being virtually pain-free within 24 hours, a state that persisted for approximately one week before mild aching recurred. The patient described a profound improvement in her ability to ambulate, climb stairs, and stand for extended periods without the previous aching or catching sensation. She attended full clinical and classroom activities comfortably and reported improved quality of life and confidence in movement. Mild soreness returned after approximately one week, but was easily managed with myofascial maintenance by her partner.

Pain remained stable at 2-3/10 for several months following the initial intervention, with no recurrence of the debilitating episodes that had previously required orthopedic evaluation. Subsequent treatments, including daily myofascial release and muscle energy techniques administered by her partner (a fellow osteopathic medical student), maintained pain at 1-2/10 without pharmacologic support [12,13]. She resumed normal daily activities and prolonged standing with minimal discomfort. No adverse events occurred.

## Discussion

This case demonstrates the potential for targeted OMT, particularly HVLA to the fibular head, to relieve chronic knee pain that might otherwise be managed through invasive means. Fibular head somatic dysfunction can arise from trauma, repetitive strain, or compensatory gait mechanics [10]. Anterior fibular head somatic dysfunction shows restricted posterior glide and can alter lateral knee tension through the biceps femoris and lateral collateral ligament attachments, contributing to local irritation and pain.

Corrective manipulation likely restored physiologic joint motion and normalized afferent proprioceptive signaling, interrupting chronic pain cycles and improving functional outcomes [14-16]. Differential considerations for chronic lateral knee pain include iliotibial band syndrome, lateral meniscal pathology, lateral collateral ligament sprain, patellofemoral pain syndrome, and proximal tibiofibular joint dysfunction. Osteopathic examination supported the latter as the primary driver of symptoms in this patient.

In this case, the combination of mechanical stress from historical obesity and systemic inflammation from endocrine and metabolic disease likely contributed to both the onset and chronicity of the patient's knee pain. Prolonged altered gait mechanics secondary to muscle imbalance and joint strain may have predisposed the fibular head to anterior displacement. The patient's comorbid hypothyroidism and diabetes, both associated with delayed soft tissue healing and glycosylation of connective tissues, further complicated her ability to recover from repetitive microtrauma [4,7,10,11]. These overlapping systemic and biomechanical factors exemplify the multifactorial nature of chronic musculoskeletal pain, highlighting the unique advantage of osteopathic assessment in addressing both structural and physiologic dimensions.

Existing literature supports OMT as a complementary approach for lower-extremity pain and joint dysfunction, including comparative benefit relative to exercise-based interventions in select knee conditions [15,17]. This case is notable for (1) the patient's youth and impending surgical candidacy, (2) immediate and durable response to OMT, and (3) successful maintenance through continued noninvasive care. For patients with metabolic or autoimmune comorbidities, OMT presents minimal systemic risk compared with repeated corticosteroid injections or surgery.

Additionally, this case underscores the importance of individualized, integrative management strategies in patients with chronic pain who may be considered poor surgical candidates due to age or systemic comorbidities. OMT aligns with preventive and functional medicine principles, offering both mechanical correction and potential modulation of autonomic tone and local circulation, particularly in underserved or resource-limited settings [16,18]. The patient's response further supports the role of osteopathic manipulation not only in acute symptom relief but also in improving long-term biomechanical stability. Further controlled studies are warranted to evaluate the efficacy, duration of benefit, and optimal frequency of HVLA treatments to the fibular head, as well as their integration with rehabilitative exercises and endocrinologic management.

## Conclusions

OMT, including HVLA directed at fibular head somatic dysfunction, may offer a safe and effective alternative to surgery for chronic knee pain in appropriately selected young adults. Recognition of fibular biomechanics and application of osteopathic manipulative principles can restore function, relieve pain, and potentially delay or diminish the need for surgical intervention in appropriately selected patients. This case demonstrates how osteopathic intervention can address both structural and systemic contributors to chronic pain. For patients with metabolic or autoimmune conditions, OMT offers a low-risk, non-pharmacologic treatment modality that may complement standard medical management and reduce the need for surgical procedures. Recognition of such cases early in care pathways could meaningfully alter long-term outcomes and reduce healthcare burden.
